# Evaluation of a multifaceted medication review in older patients in the outpatient setting: a before-and-after study

**DOI:** 10.1007/s11096-022-01531-3

**Published:** 2023-02-06

**Authors:** N.A. Zwietering, A. E. M. J. H. Linkens, P. H. M. van der Kuy, H. Cremers, N. van Nie-Visser, K. P. G. M. Hurkens, Bart Spaetgens

**Affiliations:** 1 Department of Geriatric Medicine, Laurentius Hospital, Roermond, The Netherlands; 2 Department of Internal Medicine, Geriatric Medicine, Maastricht University Medical Centre, Maastricht, The Netherlands; 3 Department of Hospital Pharmacy, Erasmus Medical Centre, Rotterdam, The Netherlands; 4 Department of Clinical Pharmacy, Pharmacology and Toxicology, Zuyderland Medical Centre, Sittard, Geleen, The Netherlands; 5 Innovation and Funding (Scientific Research), Zuyderland Medical Centre, Sittard, Geleen, The Netherlands; 6 Department of Internal Medicine, Geriatric Medicine, Zuyderland Medical Centre, PO box 5500, 6130 MB Sittard, Geleen, The Netherlands; 7 Department of Internal Medicine, Division of General Internal Medicine, Section Geriatric Medicine, Maastricht University Medical Center and Cardiovascular Research Institute Maastricht, Maastricht, The Netherlands

**Keywords:** 80 and over, Aged–Aged, Drug Utilization Review, Frail Elderly, Polypharmacy

## Abstract

**Background:**

The prevalence of medication-related emergency department visits and acute hospital admissions in older patients is rising due to the ageing of the population and increasing prevalence of multimorbidity and associated polypharmacy.

**Aim:**

To explore whether a combined medication review performed in the outpatient setting reduces the number of medication-related emergency department visits and hospital (re)admissions.

**Method:**

All consecutive patients visiting the geriatric outpatient clinic underwent a multifaceted medication review (i.e. evaluation by at least a geriatrician, and/or pharmacist and use of clinical decision support system). Subsequently, we analysed the number of, and reason for, emergency department visits, acute hospital admissions and readmissions in the year prior to and the year following the index-date (date of first presentation and medication review).

**Results:**

A multifaceted medication review reduced the number of potentially medication-related emergency department visits (38.9% vs. 19.6%, *p* < 0.01), although the total number of ED visits or acute hospital admissions per patient in the year before and after medication review did not differ.

**Conclusion:**

A multifaceted medication review performed in the outpatient clinic reduced the number of potentially medication-related emergency department visits and could therefore reduce negative health outcomes and healthcare costs.

**Supplementary Information:**

The online version contains supplementary material available at 10.1007/s11096-022-01531-3.

## Impact statements

- A multifaceted medication review performed in the outpatient setting may reduce healthcare costs associated with medication-related emergency department visits.
- Older patients could benefit from multifaceted outpatient medication review in reducing negative health outcomes.
- Health care professionals in the outpatient setting should be aware of prescribing medications that are associated with a high risk of medication-related problems in older patients.

## Introduction

The population is ageing, leading to an older and increasingly frail population [1]. This ageing of society also leads to an increase in multimorbidity and associated polypharmacy, which will have an important impact on health care, including emergency care use. Therefore, it is not surprising that the number of emergency department (ED) visits by older patients (> 65 years old) has increased considerably over the past years[2, 3]. Approximately 2.3–28.6% of these ED visits and 1.3–41% of acute hospital admissions are thought to be attributable to medication-related problems [4–6]. While the reported prevalence varies widely due to different definitions and methods of identification, the importance of recognizing medication-related ED visits and hospital admissions is undisputed, given their association with negative health outcomes and high healthcare costs [7–9]. Therefore, interventions to reduce medication-related ED visits and hospital (re)admissions have been investigated extensively, among which medication review is the most studied.

Comprehensive tools are necessary for appropriate medication review in the older population and instruments have been introduced, including the Screening Tool of Older Person’s Prescriptions and Screening Tool to Alert doctors to Right Treatment [10]. However, these tools cannot fully substitute for comprehensive geriatric and pharmacological medication review, and these methods should be combined in personalized drug treatment. Prior research on the effects of interventions to prevent medication-related problems shows a great deal of diversity in types of healthcare professionals involved (i.e. pharmacists and medical doctors or a combination of both) and types of interventions [11]. Although a recent systematic review showed that an isolated medication review has no effect on the number of hospital admissions, multiple studies claim the opposite, which might be due to the fact that medication reviews are often part of a more comprehensive intervention [12–15]. Prior research on pharmacist-led interventions showed a possible reduction in medication-related ED visits, but with great heterogeneity between studies [16]. Therefore the effects of medication review in the outpatient setting on the number of medication-related ED visits is still uncertain.

### Aim

The aim of the study was to explore whether a combined medication review performed by a geriatrician, outpatient pharmacist and clinical decision support system in the outpatient setting could reduce the number of medication-related ED visits and hospital (re)admissions.

### Ethics approval

The study was approved on June 13th 2016 by the Medical Research Ethics Committee of Zuyderland Medical Centre (study number 16-N-115) with a waiver of consent.

## Method

### Setting

This retrospective and observational study was performed in the Zuyderland Medical Centre, a large 980-bed teaching hospital in the South of the Netherlands with three main locations. It is the second largest hospital in the Netherlands and treats roughly 190,000 patients at the outpatient clinic per year.

### Data collection and analysis

Between May and July 2016, a convenience sample of all new patients who visited the outpatient clinic geriatric medicine were included (n = 200). There were no specific inclusion or exclusion criteria. We collected patient characteristics (i.e. age, sex), number of drugs, and categorized these according to ATC (Anatomical Therapeutic Chemical Classification System) codes from the electronic patient file and medication system [17]. Based on the information available during the visit, the geriatrician performed a medication review (n = 200), according to usual care (and using the national guideline) [18]. The remarks on medication review were collected from the patient’s file by the research team. Furthermore, it was intended to let an outpatient pharmacist review the patient’s medication independently in a consultation prior to the visit at the geriatrician, guided by the previously mentioned guideline. The pharmacist had access to the same information as the geriatricians. The remarks on medication review by the pharmacist were sent to the research team. However, due to logistical challenges, only 54 out of 200 medication profiles were reviewed by the pharmacist. These patients were selected randomly, based on presence of the pharmacist that day. Finally, 197 out of 200 medication profiles were assessed by the Clinical Decision Support System (CDSS) (3 patients did not consent to the digital exchange of data on medication use) (Figure S1, supplementary material). Only after the visit, the CDSS was run, so the geriatrician and outpatient pharmacist did not have the CDSS’s input for their patients. The information collected on medication review was shared in the patient’s file, accessible to all disciplines and was sent to the primary care physician to adjust the patient’s medication.

The recommendations of the geriatrician, pharmacist and reports from the CDSS were categorized by the research team in seven pre-established categories, i.e. ‘indication without medication’, ‘medication without indication’, ‘contra-indication/interaction/side-effect’, ‘dosage problem’, ‘double medication’, ‘incorrect medication’ and ‘therapeutic drug monitoring’. These categories have been used in previous research and are based on possible and common pharmacotherapeutic problems [19]. Subsequently, we analysed the number of, and reason for, ED visits the year prior to and the year following the index-date (date of medication review). Moreover, the number of, and reason for, acute hospital admissions the year prior to and the year following the index-date was analysed. Finally, we analysed 30-day and 180-day hospital readmission rates. We also identified if the reason for ED visit was potentially medication-related based on previously described adverse drug events (ADEs) [20].

### Clinical decision support system (CDSS)

The Clinical Rule Reporter (CRR) is a CDSS that was developed in the Zuyderland Medical Centre. The CDSS runs daily and reviews medication profiles of all hospitalized patients and, in addition, medication-related data of outpatient populations can be imported and reviewed. The CDSS combines patient characteristics (e.g. age and renal function) and medication-related information (e.g. dosage, interactions) to obtain specific advice based on clinical rules (i.e. algorithms). These are clearly defined rules, that include among others, the latest version of the STOPP/START criteria and utilizes triggers to identify the need to discontinue, to initiate or to reduce dosages [10, 21]. The set of clinical rules is being updated constantly based on insights from professional networks, research and guidelines [22].

### Statistical analysis

Statistical analysis of the data was performed with IBM SPSS Statistics 23. Numerical variables are reported as means (± standard deviation (SD)) and categorical variables as percentages (%). Chi-square/ Fisher’s exact test was used to analyse categorical variables and student’s *t*-tests for continuous variables. Non-parametric testing was used to analyse differences in number of ED visits, hospital admissions and length-of-hospital stay. To investigate the contribution of different medication categories to the occurrence of an ED visit or hospitalization, univariable logistic regression analyses were performed. To explore the impact of polypharmacy we performed age and sex-adjusted multivariable regression analyses. A *p*-value ≤ 0.05 was considered statistically significant.

## Results

### Overview

A total of 200 patients, who presented to the outpatient clinic geriatric medicine of which 118 (59%) were female, with a mean age of 82 (± 7) years were included. There was no difference in the median number of drugs in use before and after medication review (8.0 (IQR 5–11) vs. 8.0 (IQR 6–11), *p* = 0.153). When categorizing medication to ATC code, we found that agents acting on the renin-angiotensin system (48%), proton pump inhibitors (PPIs) (59%) and antithrombotic agents (64%) were the most frequently used. Moreover, there was frequent use of beta blocking agents (41%), vitamin D (42%), diuretics (42%), and statins (44%). Roughly 25% of the patients used sedatives, mainly benzodiazepines. Adjustments in medication profiles were carried out in 120 patients. Table 1 shows the demographics.

**Table 1:** Demographics

Characteristics	
Age. Mean (± SD)	82(± 7)
Sex	
Female, n (%)	118 (59.0)
Age class	
60–70, n(%) 71–75, n(%) 76–80, n(%) 81–85, n(%) >85, n(%)	12 (6.0) 23 (11.5) 49 (24.5) 58 (29.0) 58 (29.0)
Polypharmacy	
5–9 drugs, n(%) ≥10 drugs, n(%)	85 (42.5%) 67 (33.5%)
Drugs in use. Median (IQR)	8.0 (5–11)

### Emergency department visits

The total number of ED visits in our population did not differ significantly before and after medication review (n = 91 vs. n = 96, *p* = 0.55). However, there was a major difference in the number of potentially medication-related ED visits before and after medication review, respectively (38.9% vs. 19.6%, *p* < 0.01). Moreover, there was a trend towards less fall incidents (and associated fractures) in the after medication review group (37.4% vs. 24.0%, *p*= 0.06). On the other hand there was also a trend towards more intracranial haemorrhages (4 spontaneous and 1 post-traumatic) in the after medication review group (*p* = 0.06) (Table 2). We found no differences between the total number of unique patients with an ED visit (69 vs. 64, *p* = 0.60).

**Table 2:** Emergency department (ED) visits

Total population n = 200	Before medication review	After medication review	*p*-value
Total number of unique patients with ED visit (n)	69	64	0.6
Total number of ED visits	91	96	0.62
Potentially medication-related, n (%)	35 (38.9)	19 (19.6)	< *0.01*
Median number of ED visits per patient (IQR)	0 (0–1)	0 (0–1)	0.75
*Reasons for ED visit, n (%)*			
Electrolyte disorders	5 (5.4)	1 (1.0)	0.11
*Neurological disease*			
Transient ischemic attack/stroke	10 (11.0)	12 (12.5)	0.82
Delirium/confusion	3 (3.3)	8 (8.3)	0.21
Other	2 (2.2)	8 (8.3)	0.1
Infection/sepsis	10 (11.0)	15 (15.6)	0.4
Falls (incl. associated fractures)	34 (37.4)	23 (24.0)	*0.06*
Surgical	1 (1.1)	2 (2.1)	1
*Cardiovascular disease*			
Acute coronary syndrome	3 (3.3)	5 (5.2)	0.72
Heart failure	3 (3.3)	2 (2.1)	0.68
Cardiac arrhythmias	6 (6.6)	3 (3.1)	0.32
Pulmonary embolism	1 (1.1)	0	0.49
Pulmonary disease	1 (1.1)	4 (4.2)	0.37
Gastro-intestinal disease	2 (2.2)	1 (1.0)	0.61
*Haemorrhage*			
Intracranial	0	5 (5.2)	*0.06*
Gastro-intestinal	0	1 (1.0)	1
Urogenital	1 (1.1)	1 (1.0)	1
Other	1 (1.1)	0	0.49
Musculoskeletal disease	4 (4.4)	1 (1.0)	0.2
Urological	2 (2.2)	2 (2.1)	1
Other	2 (2.2)	2 (2.1)	1

### Hospital admission

There were no differences in the number of unique patients admitted (n = 39 vs. n = 55, *p*= 0.10) or in the total number of admissions (n = 55 vs. n = 72, *p*= 0.09).

Focusing on reasons for admission there were no statistical differences between the before medication review group, compared to the after medication review group. Of particular interest were electrolyte disorders, falls and haemorrhages, although these also did not differ significantly (Table 3).

**Table 3:** Hospital admissions

Total population n = 200	Before medication review		After medication review		*p*-value
Total number of unique patients admitted (n)	39		55		0.1
Number of patients with repeat admission, n (%)	9 (22.0)		13 (23.2)		1
Total number of admissions (n)	55		72		0.09
Number of admissions per patient, n (%)	0	145 (72.5)	0	128 (64.0)	0.09
	1	39 (19.5)	1	55 (27.5)	0.08
	2	10 (5.0)	2	15 (7.5)	1
	3	4 (2.0)	3	2 (1.0)	0.23
	4	1 (0.5)	4	0	0.41
	5	1 (0.5)	5	0	0.41
Median number of admissions per patient (IQR)	0.0 (0–0)		0.0 (0–1)		0.1
Median length of in-hospital stay (days) (IQR)	5 (3–11)		6 (3–9)		0.14
*Rate of readmission, n (%)*					
30 day	4 (7.3)		2 (2.8)		0.23
180 day	11 (20.0)		13 (18.0)		0.64
*Reasons for hospital admission, n (%)*					
Electrolyte disorders	2 (3.6)		–		0.19
*Neurological disease*					
Transient ischemic attack/stroke	9 (16.4)		10 (13.9)		0.8
Delirium/confusion	1 (1.8)		3 (4.2)		0.63
Neuritis vestibularis	–		1 (1.4)		1
Infection/sepsis	11 (20)		18 (25)		0.53
Falls (incl. associated fractures)	7 (12.7)		13 (18.1)		0.47
*Cardiovascular disease*					
Acute coronary syndrome	4 (7.3)		2 (2.8)		0.4
Heart failure	4 (7.3)		1 (1.4)		0.17
Cardiac arrhythmias	4 (7.3)		6 (8.3)		1
Pulmonary embolism	2 (3.6)		0		0.19
Peripheral vascular disease	1 (1.8)		1 (1.4)		1
Pulmonary disease	4 (7.3)		6 (8.3)		1
Gastro-intestinal disease	1 (1.8)		1 (1.4)		
*Haemorrhage*					
Intracranial	0		5 (6.9)		0.07
Gastro-intestinal	0		1 (1.4)		1
Urogenital	1 (1.8)		1 (1.4)		1
Musculoskeletal disease	1 (1.8)		1 (1.4)		1
Kidneyinjury	2 (3.6)		0		0.19
Inflammatory disease	1 (1.8)		0		0.43
Other	0		2 (2.8)		0.51

### Medication use

In the patients that presented to the ED, there were no differences in medication use before and after medication review. These results and breakdown into different ATC categories are summarized in Table 4.

**Table 4:** Medication use in patients comparing ED visits before and after medication review

No. of patients (%) ATC code	ED visit BMR (n = 69)	ED visit AMR (n = 64)	p-value
A10A (insulins and analogues)	6 (8.7)	3 (4.7)	0.36
B01 (antithrombotic agents)	47 (68.1)	44 (68.8)	0.94
C01 (cardiac therapy)	19 (27.5)	19 (29.7)	0.78
C02 (antihypertensive drugs)	2 (2.9)	1 (1.6)	0.60
C03 (diuretics)	30 (43.5)	24 (37.5)	0.48
C07 (beta blocking agents)	26 (37.7)	27 (42.2)	0.60
C08 (Ca-channel blockers)*	15 (21.7)	14 (21.9)	0.98
C09 (agents acting on RAS)#	37 (53.6)	26 (40.6)	0.13
G (genito-urinary system)	10 (14.5)	9 (14.1)	0.94
N02A (opioids)	15 (21.7)	10 (15.6)	0.37
N04 (anti-Parkinson drugs)	4 (5.8)	1 (1.6)	0.20
N05A (antipsychotics)	5 (7.2)	5 (7.8)	0.90
N05C (hypnotics and sedatives)	18 (26.1)	16 (28.1)	0.53
N06A (antidepressants)	8 (11.6)	13 (20.3)	0.17
N06D (dementia drugs)	4 (4.4)	3(4.7)	0.77

*BMR *  before medication review, *AMR* after medication review

*Ca  calcium, #*RAS * renin-angiotensin system

In patients that were hospitalized we found that the use of diuretics was significantly higher in patients that had an acute hospital admission before medication review (64.1% vs. 38.2%, *p* = 0.01) and that they tended to use more anti-Parkinson drugs (10.3% vs. 1.8%, *p* = 0.07). These and results for other ATC-categories were summarized in Table 5.

**Table 5:** Medication use in patients comparing acute hospital admissions before and after medication review

ATC code	No. of patients (%)		*p*-value
	Acute hospital admission BMR (n = 39)	Acute hospital admission AMR (n = 55)	
A10A (insulins and analogues)	6 (15.4)	4 (7.3)	0.21
B01 (antithrombotic agents)	28 (71.8)	38 (69.1)	0.78
C01 (cardiac therapy)	13 (33.3)	15 (27.3)	0.53
C02 (antihypertensive drugs)	–	–	
C03 (diuretics)	25 (64.1)	21 (38.2)	*0.01*
C07 (beta blocking agents)	19 (48.7)	26 (47.3)	0.89
C08 (Ca-channel blockers)*	8 (20.5)	12 (21.8)	0.88
C09 (agents acting on RAS)#	22 (56.4)	23 (41.8)	0.13
G (genito-urinary system)	6 (15.4)	9 (16.4)	0.90
N02A (opioids)	7 (17.9)	12 (21.8)	0.65
N04 (anti-Parkinson drugs)	4 (10.3)	1 (1.8)	*0.07*
N05A (antipsychotics)	3 (7.7)	3 (5.5)	0.66
N05C (hypnotics and sedatives)	12 (30.8)	14 (25.5)	0.52
N06A (antidepressants)	5 (12.8)	12 (21.8)	0.26
N06D (dementia drugs)	3 (7.7)	3 (5.5)	0.66

*BMR * before medication review, *AMR* after medication review

*Ca calcium, #*RAS * renin-angiotensin system

Univariable regression analyses of medication categories associated with ED visits or hospitalization, before and after medication review are presented in Table S2 (supplementary material). As such, we found that diuretics (OR 3.09, 95% CI 1.49–6.40, *p* = 0.002) and anti-Parkinson drugs (OR 6.02, 95% CI 1.29–28.11, *p* = 0.02) were independently associated with hospitalization in the before medication review group and that dementia drugs tended to significance (OR 4.39, 95% CI 0.85–22.64, *p* = 0.07). In the after medication review group, no medication categories were significantly associated with ED visits or hospitalization (Table S2). 

Age- and sex-adjusted multivariable regression analyses showed that polypharmacy was independently associated with hospitalization and that the OR was slightly lower in the after medication review group compared to the before medication review group (OR 2.69, 95% CI 1.11–6.47, vs. OR 3.52, 95% CI 1.16–10.63, respectively).

## Discussion

### Key findings and interpretation

This study demonstrates the impact of a multifaceted medication review in older patients in the outpatient setting in reducing (medication-related) ED visits and hospital admissions. As such, we have shown an almost 20% absolute decrease of potentially medication-related ED visits, although the total number of ED visits was not altered. This is in line with previous numbers estimating a reduction of 27% (ranging from 45% reduction to 3% increase in visits) [13]. Additionally, we found no differences between hospital admissions before and after medication review.

The significant reduction in potentially medication-related ED visits after a multifaceted medication review in the outpatient setting was not surprising, since a major goal of the intervention was to prevent ADEs. As such, we found (although not statistically significant) a reduced number of falls incidents and associated fractures that lead to ED visits, suggesting a potential mechanism of action. In line with this, we found in the univariable analysis that diuretics, antidepressants and dementia drugs were associated with hospitalization before medication review, but this was no longer the case in the univariable analysis after medication review. The underlying diseases associated with some of these drugs, in particular dementia and chronic heart failure are obviously predictors of hospitalization on their own [23, 24]. Diuretics in particular are known in the top 10 of potentially preventable ADEs such as electrolyte disturbances and falls [25–27]. Finally, we also have shown that the OR of polypharmacy as contributing factor to hospital admissions slightly decreased in the after medication review group. Nevertheless, it still remained an important contributing factor to hospital admission.

In the light of the above, it was surprising that neither the total number of ED visits nor the total number of hospital admissions were reduced in the year after medication review. Several explanations for these negative findings may be given. First, apparently while patients were selected in the outpatient clinic of geriatric medicine, we may have included the most vulnerable population at risk for adverse outcome, as they might have been referred for a specific reason to this outpatient clinic (i.e. falling or cognitive decline). The median of 8 drugs in use further supports this vulnerability and it is unlikely that medication review had led to using less than 5 drugs in this population. Second, by including this vulnerable and older population (mean age 82 years), numerous reasons for ED visits or hospitalization may occur. Prior research indicated that age (≥ 65 years), number of medications and comorbidity were all associated risk factors for acute hospital admission [28].

The negative finding of not being able to reduce ED visits or hospital admissions is in line with the systematic review and meta-analysis of Huiskes et al., that showed no effect of medication review on clinical outcomes [29]. It should be stated however, that the mean follow-up in the research by Huiskes et al. was only 5.2 months. This follow-up period could have affected their results, because in our study most patients were admitted 6–12 months after medication review.

This study showed no difference in the rate of 30-day and 180-day readmission. There is previous literature, with low-quality evidence, suggesting an impact of pharmacist-led medication review on medication-related readmissions [30, 31]. Ravn-Nielsen et al. however showed that the combination of medication review, motivational interviewing with the patient and follow-up in primary care, had a significant effect on lowering the rates of readmission at 30 and 180 days [31]. This suggests that solely an in-hospital medication review is not sufficient to have an effect on readmission rates, but in particular clear communication with and smooth transfer to primary care is of importance. In our study there was written communication to primary care, but due to the nature of the study, there was no control on the continued adherence to advice following from medication review. Therefore, it is hypothetically possible that less favourable medication was represcribed in primary care during follow-up that could have led to medication-related readmission.

### Strengths and weaknesses

The strengths of this study are that we analysed the effect of a multifaceted medication review in the geriatric outpatient clinic, without any further selection criteria, whereas most of previous literature focused on hospitalized patients [6, 12, 13]. By doing so, we were able to include the most vulnerable and therefore also most at risk population. Moreover, our follow-up was 12 months, which is longer than most studies on the effects of medication review. As such, negative findings are also of interest, given the fact that if any effect fades between 6 and 12 months, one might focus on other interventions that may result in longer-lasting effects, such as continuous and periodic medication reviews by general practitioners and clinical pharmacists.

This study also has several limitations. First, it is limited by its retrospective and observational design, lack of standardisation and relatively small number of patients enrolled. Second, due to logistical challenges, only about a quarter of the patients enrolled had pharmacist involvement and although this did not alter the results, this was not as intended and as such may have introduced bias. Third, while we present a very vulnerable population, we were unable to adjust for frailty status. Finally, another limitation is the fact that the outcome measures in our study may have not been appropriate to fully the capture the benefit of our intervention and that our study may be underpowered for several outcomes. That being said, to avoid adverse health outcomes, such as (medication-related) ED visits or hospitalizations is very important from the patient perspective, while reducing this number is very important from a societal and economic perspective.

### Further research

In further research we will perform a randomized trial where we analyse the effects of medication review on outcome (mortality, adverse events, length-of stay) of acutely hospitalized older patients.

## Conclusion

A multifaceted medication review performed in the outpatient clinic reduced the number of potentially medication-related emergency department visits and could therefore reduce negative health outcomes and healthcare costs.

## Electronic supplementary material

Below is the link to the electronic supplementary material.

Supplementary Material 1
