# Supplementary material for: Evaluation of a multifaceted medication review in older patients in the outpatient setting: a before-and-after study
Source: Int J Clin Pharm. 2023 Feb 6;45(2):483–90. doi: 10.1007/s11096-022-01531-3 (PMC10147805; doi:10.1007/s11096-022-01531-3)
Supplement: Supplementary file 1 — Supplementary Material 1 [file 11096_2022_1531_MOESM1_ESM.docx]

**Supplementary material**

*Figure S1. Schematic overview of data collection*

*Table S2. Univariable logistic regression analyses to explore the impact of different medication categories for the occurrence of ED visits and hospitalization before and after medication review*

| **Variables** | **Univariable analysis ED BMR** | | **Univariable analysis ED AMR** | | **Univariable analysis hospital admission BMR** |  | **Univariable analysis hospital admission AMR** |  |
| --- | --- | --- | --- | --- | --- | --- | --- | --- |
|  | B (95% CI) | P | B (95% CI) | P | B (95% CI) | P | B (95% CI) | P |
| Age, years | 0.97 (0.93 - 1.01) | 0.12 | 1.01 (0.96 - 1.06) | 0.75 | 0.98 (0.93 - 1.03) | 0.44 | 1.02 (0.98 - 1.07) | 0.33 |
| Female sex | 0.78 (0.43 – 1.41) | 0.41 | 0.78 (0.42 - 1.46) | 0.43 | 0.60 (0.29 - 1.20) | 0.15 | 0.85 (0.46 - 1.55) | 0.59 |
| Polypharmacy (yes) | 1.38 (0.68 – 2.79) | 0.37 | 2.70 (1.13 - 6.46) | *0.025* | 3.29 (1.11 - 9.80) | *0.032* | 2.46 (1.11 - 5.45) | *0.027* |
| **Use of any of the following drugs(yes)** | | | | | | | |  |
| A10A (insulins and analogues) | 1.29 (0.44 - 3.79) | 0.64 | 0.96 (0.29 - 3.14) | 0.94 | 3.07 (1.02 - 9.22) | 0.045 | 0.51 (0.14 - 1.87) | 0.31 |
| B01 (antithrombotic agents) | 1.32 (0.71 - 2.44) | 0.38 | 1.37 (0.70 - 2.65) | 0.36 | 1.55 (0.72 - 3.34) | 0.26 | 1.36 (0.72 - 2.56) | 0.34 |
| C01 (cardiac therapy) | 1.40 (0.71 - 2.74) | 0.33 | 1.32 (0.65 - 2.70) | 0.44 | 1.87 (0.87 - 4.01) | 0.11 | 1.63 (0.83 - 3.21) | 0.16 |
| C02 (antihypertensive drugs) | 1.27 (0.21 - 7.81) | 0.79 | n/a |  | n/a |  | 0.52 (0.06 - 4.78) | 0.57 |
| C03 (diuretics) | 1.10 (0.61 - 1.98) | 0.76 | 0.80 (0.43 - 1.52) | 0.50 | 3.09 (1.49 - 6.40) | 0.002 | 0.76 (0.41 - 1.40) | 0.38 |
| C07 (beta blocking agents) | 0.81 (0.45 - 1.47) | 0.49 | 1.43 (0.76 - 2.67) | 0.27 | 1.48 (0.73 - 2.99) | 0.28 | 1.08 (0.59 - 1.96) | 0.82 |
| C08 (Ca-channel blockers)* | 1.38 (0.66 - 2.86) | 0.39 | 1.34 (0.62 - 2.90) | 0.46 | 1.18 (0.49 - 2.82) | 0.72 | 1.38 (0.65 - 2.89) | 0.40 |
| C09 (agents acting on RAS)# | 1.41 (0.79 - 2.53) | 0.25 | 0.71 (0.38 - 1.33) | 0.28 | 1.52 (0.75 - 3.08) | 0.24 | 0.65 (0.35 - 1.18) | 0.15 |
| G (genito-urinary system) | 1.14 (0.49 - 2.64) | 0.77 | 1.38 (0.58 - 3.29) | 0.47 | 1.21 (0.45 - 3.24) | 0.70 | 1.07 (0.45 - 2.54) | 0.87 |
| N02A (opioids) | 1.38 (0.66 - 2.86) | 0.39 | 1.34 (0.62 - 2.90) | 0.46 | 0.96 (0.39 - 2.37) | 0.92 | 0.75 (0.34 - 1.65) | 0.47 |
| N04 (anti-Parkinson drugs) | 2.63 (0.57 - 12.08) | 0.22 | 0.43 (0.05 - 3.65) | 0.44 | 6.02 (1.29 - 28.11) | 0.022 | 0.34 (0.04 - 2.92) | 0.33 |
| N05A (antipsychotics) | 2.48 (0.64 - 9.56) | 0.19 | 1.34 (0.32 - 5.54) | 0.69 | 2.15 (0.51 - 9.02) | 0.29 | 2.80 (0.73 - 10.79) | 0.14 |
| N05C (hypnotics and sedatives) | 0.97 (0.50 - 1.88) | 0.92 | 0.93 (0.46 - 1.89) | 0.84 | 1.30 (0.60 - 2.80) | 0.50 | 1.13 (0.58 - 2.20) | 0.72 |
| N06A (antidepressants) | 0.48 (0.21 - 1.13) | 0.09 | 1.41 (0.65 - 3.06) | 0.39 | 0.62 (0.22 - 1.71) | 0.35 | 1.25 (0.59 - 2.67) | 0.56 |
| N06D (dementia drugs) | 3.97 (0.71 - 22.24) | 0.12 | 2.73 (0.53 - 13.96) | 0.23 | 4.39 (0.85 - 22.64) | 0.077 | 2.18 (0.43 - 11.11) | 0.35 |

*ED = emergency department BMR= before medication review, AMR=after medication review*
**Ca = calcium #RAS = renin-angiotensin system*
